# A Rare Case of Abdominal Wall Skeletal Muscle Metastasis From Adenocarcinoma of the Pancreatic Head

**DOI:** 10.7759/cureus.41470

**Published:** 2023-07-06

**Authors:** Brittney Shupp, Hammad Liaquat, Zarian Prenatt, Lisa Stoll, Ayaz Matin

**Affiliations:** 1 Internal Medicine, St. Luke's University Health Network, Bethlehem, USA; 2 Gastroenterology, St. Luke's University Health Network, Bethlehem, USA; 3 Pathology, St. Luke's University Health Network, Bethlehem, USA

**Keywords:** pancreatic head, adenocarcinoma, skeletal muscle metastasis, metastasis, medical oncology, surgical oncology, pancreatic head adenocarcinoma, gastroenterology

## Abstract

Pancreatic cancer can be aggressive and commonly metastasizes to various organs. Most commonly, pancreatic cancer metastasizes to the lung, liver, bones, and peritoneum, but very rarely does it spread to the abdominal wall or skeletal muscle. In this case, we discuss a patient who initially presented with weight loss and jaundice from a pancreatic head adenocarcinoma that later metastasized to the rectus abdominis muscle. A 63-year-old female presented with jaundice and weight loss. CT imaging revealed a 2.8 cm pancreatic head mass with pancreatic and biliary ductal dilation. Carbohydrate antigen 19-9 (CA 19-9) level was also found to be elevated to 1810 U/mL. An endoscopic ultrasound-guided biopsy was later performed and confirmed pancreatic adenocarcinoma. The patient underwent a Whipple pancreatoduodenectomy following initial treatment with neoadjuvant FOLFIRINOX chemotherapy. Following the Whipple procedure, she received adjuvant chemotherapy and subsequent imaging revealed no recurrence and decreased CA 19-9 level to 46 U/mL. Eight months afterward, the patient presented once again with lower abdominal pain. Repeat CA 19-9 level was found to have increased to 1503 U/mL. Repeat positron emission tomography scan imaging was performed and showed a 4.7 cm left rectus abdominis muscle mass. The mass was later biopsied, and pathology revealed recurrent, metastatic pancreatic adenocarcinoma. The patient was restarted on chemotherapy with paclitaxel and gemcitabine leading to a reduction in tumor size and CA 19-9 levels of 135 U/mL. However, surgical resection was later pursued due to increased tumor size only four months later. At this time, limited literature is available reporting the occurrence of pancreatic cancer metastasizing to the abdominal wall. Upon literature review, only five cases have been reported to date, and only two of the cases involved the skeletal muscle. Our rare case is the first-time documentation of rectus abdominis metastasis from pancreatic adenocarcinoma arising from the pancreatic head.

## Introduction

Pancreatic cancer is a challenging disease that has a very low survival rate considering its early and rapid metastasis, often present at the time of diagnosis [1]. In these cases, metastasis most commonly spreads to the lung, liver, bones, and peritoneum [2]. Both abdominal wall and skeletal muscle metastasis are extremely rare in association with pancreatic cancer. They can easily be misdiagnosed as primary soft tissue sarcoma without a biopsy [3]. In this case, we discuss a rare case of rectus abdominis muscle metastasis from pancreatic cancer [4].

## Case presentation

**Figure 1:**
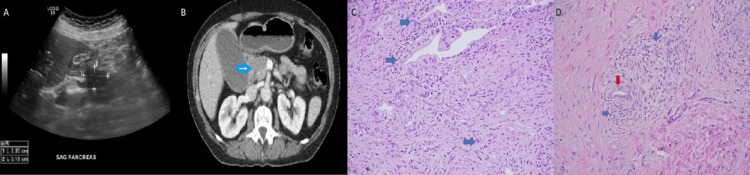
Right upper quadrant ultrasound showing a 2.9 cm pancreatic head mass (A), which is confirmed on CT of the abdomen and pelvis with contrast (arrow) in the absence of any metastatic lesions (B). Hematoxylin and eosin (H&E) stain (20x) of pancreatic tissue from pancreatic endoscopic ultrasound-guided biopsy (C) showing adenocarcinoma (arrows) and nuclear pleomorphism (4:1 ratio), incomplete glands, and few single cells in a background of desmoplastic stroma. H&E stain (20x) of pancreatic tissue from Whipple procedure (D) showing perineural invasion by pancreatic adenocarcinoma. The blue arrow indicates nerve while the red arrow indicates adenocarcinoma.

A 63-year-old Caucasian female presented with a 15-pound weight loss and jaundice. On initial presentation, vital signs were stable and laboratory testing revealed a total bilirubin of 11 mg/dL, aspartate aminotransferase/alanine aminotransferase of 138/56 U/L, and alkaline phosphatase of 282 U/L. Right upper quadrant ultrasound and computed tomography (CT) imaging showed a mass in the pancreatic head measuring 2.8 cm with ductal dilation (Figures 1A, 1B). Follow-up serum carbohydrate antigen 19-9 (CA 19-9) was elevated at 1810 U/mL. Endoscopic ultrasound-guided biopsy confirmed pancreatic adenocarcinoma (Figures 1C, 1D). At the time of initial diagnosis, extensive imaging revealed no metastatic disease. Therefore, the patient underwent neoadjuvant FOLFIRINOX chemotherapy followed by an open Whipple pancreatoduodenectomy during which perineural and lymphovascular invasion was noted. Following surgical resection, the patient completed adjuvant chemoradiation with capecitabine.

**Figure 2:**
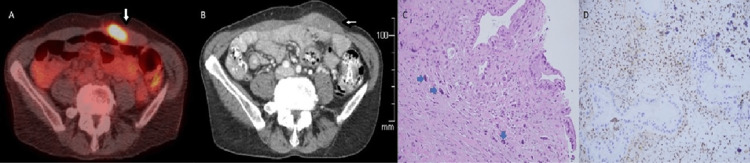
PET CT scan showing asymmetric thickening of the left rectus abdominis muscle with a 4.7 cm focally intense FDG activity (arrow) in (A). CT of the abdomen with contrast shows decreased size of metastases (now measuring 1.6 cm in size) in the persistently thickened left rectus abdominis muscle (arrow) after chemotherapy in (B). Hematoxylin and eosin stain (20x) from the biopsy of rectus abdominis mass seen on PET scan, which shows adenocarcinoma involving atrophic skeletal muscle fibers (nuclear bags – blue arrows) in (C). DPC4 immunohistochemistry stain (40x) from skeletal muscle tissue showing loss of expression within the adenocarcinoma, consistent with pancreatic origin while background stromal cells maintain DPC4 expression (D). PET; positron emission tomography; FDG: fluorodeoxyglucose.

Follow-up studies revealed a decreased CA 19-9 level to 46 U/mL and no evidence of recurrence on CT imaging. Unfortunately, only eight months after, the patient developed left lower quadrant pain and an increase in her CA 19-9 to 1503 U/mL. A follow-up positron emission tomography (PET) scan showed a mass in the left rectus abdominis muscle measuring 4.7 cm. A biopsy was completed and pathology confirmed recurrent and now metastatic pancreatic adenocarcinoma (Figure 2A). No tumor in the remaining pancreatic tissue was seen. Chemotherapy was restarted with paclitaxel and gemcitabine resulting in a decrease in the size of the lesion to 1.6 cm and the CA 19-9 level to 135 U/ml (Figure 2B). Following the completion of two cycles of chemotherapy, a PET scan confirmed improvement and demonstrated no evidence of disease to justify surgical resection or radiation.

Four months following chemotherapy, a repeat PET scan revealed recurrence in the left rectus muscle at the initial site (size 1.3 cm). Surgical excision of the rectus abdominis mass was performed and histological analysis was consistent with metastatic pancreatic adenocarcinoma (Figures 2C, 2D). CA 19-9 trended down to normal levels within a few weeks and subsequent imaging did not show recurrence or evidence of new metastasis.

## Discussion

In the United States, pancreatic cancer is the fourth leading cause of cancer-related death and seventh worldwide [5]. It is extremely aggressive and has a high potential to be advanced once diagnosed [1]. Pancreatic cancer most commonly spreads to the liver (60%), lung and perineum (30%), and bone and adrenals (10%) [6]. Conversely, muscle tissue metastasis occurs in less than 1% of cases paralleling the incidence of abdominal wall metastasis [1,2]. When generally considering metastatic carcinoma, skeletal muscle metastasis is uncommon (prevalence of 0.03% to 17.5%), but such involvement in pancreatic cancers is rare [2,7,8]. To our knowledge, only five documented cases of pancreatic cancer have reported metastasis to the abdominal wall and only two cases have reported metastasis to skeletal muscle (peroneal muscles and trapezius) [1,2,5,8-12]. Our case differentiates itself as being the only case to report pancreatic metastasis to the skeletal muscle of the abdominal wall, specifically the rectus abdominis.

The pancreas is anatomically divided into three parts: the head, body, and tail. Most commonly, pancreatic cancer involves the pancreatic head with the remaining 20-25% of cases involving the body and tail [13]. In comparison to the documented cases of skeletal muscle and abdominal wall metastasis, our case is distinguished by the cancer being confined to the head as opposed to the body or tail. Cancers of the head of the pancreas typically present with painless jaundice due to common bile duct obstruction, unlike tumors of the body and tail, which can have vague signs and symptoms. Measurement of CA 19-9 can help as a diagnostic and/or prognostic marker for pancreatic cancers. More importantly, considering the 66-92% recurrence rate of pancreatic cancer following the first two years of curative treatment, CA 19-9 surveillance aids serial imaging in the detection of cancer recurrence, such as in this case [14].

Like other forms of carcinoma, pancreatic cancer metastasizes via lymphatic, transperitoneal, or hematogenous spread [1]. In documented cases of abdominal wall metastasis, the most described mode of transmission is through postoperative abdominal wall seeding [1]. In patients with prior abdominal surgery, such as pancreatic excision, it can be difficult to delineate the etiology of metastasis being true metastasis versus a surgical complication. In these cases, analyzing the clinical context and the anatomy is imperative to discern the true cause. In many of the reported cases, the sites of metastasis have been identified at laparoscopic scars, making direct surgical seeding the unfortunate but likely culprit. In the context of our case, the patient presented with rectus abdominis metastasis eight months after the Whipple procedure. However, the site of metastasis was in a region that was distinctly different than the site of surgical operation making true metastasis the top differential. Likewise, the other documented cases of true metastasis were similar in that the site of metastasis was the umbilicus or skeletal muscle but outside of the abdominal wall [1,2,8].

Despite the location or means of metastasis, treatment of metastatic pancreatic cancer remains a challenge with the five-year survival rate being 2% [15]. Even in cases of resectable disease, prognosis remains poor with a 25-30% five-year survival rate in node-negative and 10% in node-positive tumors post-resection [15]. Current treatment options, including chemotherapy or radiation, are available for the treatment of common pancreatic metastasis sites, including the lung, liver, and bones. Due to the rarity of this skeletal muscle metastasis, more data are necessary to determine the prognosis and appropriate treatment options for cases of pancreatic cancer with skeletal muscle metastasis.

## Conclusions

We presented a rare case of rectus abdominis muscle metastasis from pancreatic cancer. Pancreatic cancer is a highly aggressive disease with a low survival rate, often presenting with early and rapid metastasis. While metastasis to the lung, liver, bones, and peritoneum is more common, metastasis to the abdominal wall and skeletal muscle is extremely rare. Our case adds to the limited literature on skeletal muscle metastasis from pancreatic cancer and highlights the importance of considering metastatic disease in atypical locations. Treatment of metastatic pancreatic cancer remains challenging, with limited treatment options and poor prognosis. Further research is needed to better understand the prognosis and appropriate management of cases with skeletal muscle metastasis from pancreatic cancer.
